# A controlled before-after study to evaluate the effect of a clinician led policy to reduce knee arthroscopy in NSW

**DOI:** 10.1186/s12891-018-2043-5

**Published:** 2018-05-16

**Authors:** H. Y. Chen, I. A. Harris, K. Sutherland, J-F. Levesque

**Affiliations:** 1 Bureau of Health Information, Level 11, 67 Albert Avenue, Chatswood, NSW 2067 Australia; 2 Whitlam Orthopaedic Research Centre, Ingham Institute for Applied Medical Research, South Western Sydney Clinical School, University of NSW, High St, Kensington, NSW 2052 Australia; 3 Centre for Primary Health Care and Equity, University of NSW, Sydney, NSW Australia; 4 Agency for Clinical Innovation, Level 4, 67 Albert Avenue, Chatswood, NSW 2067 Australia

**Keywords:** Performance and evaluation, Quality and safety, Hospitals, Clinical guidelines

## Abstract

**Background:**

Clinical evidence shows knee arthroscopy has little benefit for degenerative conditions and considerable variation in the incidence of knee arthroscopy in Australia has been identified. This study aimed to evaluate a clinician-led evidence-based policy which was implemented in one local health district in New South Wales (NSW) in 2012 to reduce the use of knee arthroscopy for patients aged 50 years or over.

**Methods:**

Trends in rates and volume of knee arthroscopy for patients 50 years or over in NSW between 2004 and 2015 by district were examined. Changes at four hospitals that adopted the policy were assessed by a quasi-experimental before and after study design with control groups, using the generalised estimating equations (GEE) Poisson model. Each case hospital was matched with four control hospitals in terms of the volume of knee arthroscopy surgeries performed in the five years prior to the intervention.

**Results:**

Between 2004 and 2015, the number of knee arthroscopies in NSW initially increased and then decreased after 2011, with considerable variation across districts. While an overall reducing trend in NSW was observed between 2011 and 2015 (39%), a 58% reduction (95% CI: 55–62%) was found in the intervention district, including the private sector, being the greatest reduction found in all districts. The GEE Poisson results show that, compared with control hospitals, the number of knee arthroscopy was significantly reduced by 56% (95% CI: 11%–79%) at four hospitals that adopted the policy during the follow-up period (*p* = 0.02).

**Conclusions:**

Clinicians in one local health district initiated a policy to restrict knee arthroscopy for patients aged 50 years or over, which may explain the greater reduction seen in that district compared to all others, despite an overall decrease noted in the state. A significant reduction found at intervened hospitals proved the effect of the policy, suggesting that the implementation of a simple clinical governance process may help reduce inappropriate surgery.

**Electronic supplementary material:**

The online version of this article (10.1186/s12891-018-2043-5) contains supplementary material, which is available to authorized users.

## Background

Clinical variation has been extensively documented across a wide range of specialties in many healthcare systems. J. Alison Glover was a pioneer in publishing a seminal study on tonsillectomy rates in 1938 [1] and around forty years later, Wennberg and Gittelsohn conducted work that culminated in the highly influential Dartmouth Atlas of Clinical Variation [2]. Some of their early work highlighted the role of clinical leadership in discontinuing procedures or low value care. “*The drop, we subsequently learned, occurred because of conscious change in treatment policy among local physicians …. Upon learning of the high rate in their area, two physicians... undertook an active review process that led to the rapid decline of their use of the procedure”* [2].

The Dartmouth Atlas catalysed efforts to explore clinical variation across many healthcare systems and focused attention on identifying treatments and procedures that are overused or provided to patients despite insufficient evidence about their efficacy and effectiveness [3–5].

Knee arthroscopy is a commonly performed orthopaedic surgical procedure, with approximately one million performed annually in the USA [6] and over 70,000 annually in Australia [7]. Significant geographic variation has been demonstrated in the rates of knee arthroscopy internationally [8] and within Australia [3]. This may represent variation in surgeon preferences or ‘supply-sensitive care’ –variation that arises not in response to evidence but due to availability of resources such as doctors or hospital beds [9].

Around 55% of knee arthroscopies in New South Wales (NSW), Australia, are performed for people aged 50 years or over, in whom degenerative changes such as osteoarthritis and meniscal tears are common [10–12]. The correlation between knee symptoms (e.g. pain, stiffness and mechanical symptoms) and the presence of meniscus tears and degenerative changes is weak. Population studies have shown that knee symptoms are not more likely in patients with meniscal tears, and that most patients with meniscus tears do not have knee pain [10, 11].

The evidence on knee arthroscopy for degenerative conditions has been accumulating for many years [13–16] and has been recently summarised in three systematic reviews [17–19] and a combined systematic review and practice guideline [20], yet observational studies using administrative data have shown that the rates of knee arthroscopy are not falling significantly, particularly for patients with degenerative conditions [21, 22]. One systematic review concluded that this surgery had no clinically important benefit in middle aged or older populations, regardless of their osteoarthritis status and found that knee arthroscopy surgery was associated with risk of harm [18]. A recent review pointed out that compared with conservative management strategies, patients who undergo knee arthroscopy do not have important benefits in pain or function over the long term [19]. Current guidelines make “a strong recommendation against the use of arthroscopy in nearly all patients with degenerative knee disease” and conclude that “further research is unlikely to alter this recommendation” [20].

In 2011, the orthopaedic department head covering two public hospitals in South Western Sydney Local Health District (SWSLHD, one of 16 districts in NSW) suggested a policy change in response to increasing evidence against the use of arthroscopy in degenerative knees, particularly affecting the middle aged and older people [13, 16]. As of 2012, the policy change restricted knee arthroscopy to patients aged less than 50 years unless approved by the department head. For patients aged 50 or over, cases presenting for knee arthroscopy were reviewed by admissions staff and forwarded to the department head. The department head reviewed but did not refuse any procedures that were submitted for surgery. The intervention involved education of the surgeons and the insertion of an extra step in the process, but not refusal to perform the surgery. This method was chosen due mainly to its simplicity and because of experience of previous success using this method to reduce the use of bone morphogenetic protein intra-operatively. The decline in case numbers was due to a lower number of patients recommended for surgery, not due to cancellation of those cases. Patients already on the waiting list for knee arthroscopy at the time of the intervention were not removed from the list. The maximum wait for surgery in NSW is 12 months.

A third public hospital adopted the policy in late 2012, and, in 2013, the orthopaedic surgeons at a fourth hospital agreed to participate. The other (fifth) orthopaedic department did not participate in the policy based on their perception of the effectiveness of the procedure in degenerative knees. While four of the five public hospitals performing knee arthroscopy in SWSLHD participated, no private hospitals were involved in the policy, although the department members involved also worked in private hospitals.

This paper describes changes in the rate and volumes of knee arthroscopy following this natural experiment. It reports trends in the volume and rate of knee arthroscopies in the state of NSW, grouped by Local Health District (LHD). The impact of policy was assessed by comparing the reductions in knee arthroscopy in SWSLHD with those in other districts and also the reductions in the four public hospitals that adopted the policy versus matched control hospitals.

## Methods

### Data

We used the Admitted Patient Data Collection including records for admitted patients from both public and private hospitals in NSW (population 7.6 million at the end of 2015), between January 1, 2004 and September 30, 2016 [23]. There are more than 220 public hospitals and health services in NSW which provide free health care to Australian citizens and permanent residents. People with private health insurance can choose to go to private hospitals (*n* = 190+). While public hospitals cover a full range of services, the majority of elective surgeries occurred in private hospitals [24]. Private hospital data after June 30, 2015 were incomplete when the analyses was conducted. The complete data in 2015 are now included in Additional file 1: Table S3, along with the overall reduction in arthroscopies between the 12 months to December 31, 2011 and the 12 months to June 30, 2017 (most recent data available). Procedure codes for knee arthroscopy are provided in Additional file 1: Table S1. Procedures associated with ligament reconstruction were excluded. Admitted patients with a knee arthroscopy procedure code in the principal or any secondary procedures between 2004 and 2016 were identified. The number of knee arthroscopies by public/private sector, and by the location of surgery (local: patients received surgery at public hospitals at their residential LHD/outflow: patients received surgery in other LHD’s public hospitals) are presented in Additional file 1: Table S2 with estimated mid-year population for the same age group. As the policy only focus on aged 50 years or over, this study only presented and discussed the results for this age group.

#### Intervention

A policy was introduced in participating hospitals that involved a clinical governance process requiring department head approval for knee arthroscopy surgery for patients aged 50 years or older. The evidence was presented at a department meeting in 2011 prior to the initial decision by the first two hospitals. No further surgeon education was performed. Additionally, a letter was written to all primary care providers (general practitioners) in the LHD explaining the evidence against knee arthroscopy in this group and against unnecessary investigations (MRI).

#### Ethical consideration

According to the policy activities that constitute research at the NSW Ministry of Health, this work met criteria for operational improvement activities exempt from ethics review.

#### Analysis

Rates of knee arthroscopy in people aged 50 and over were estimated for each calendar year (i.e., the number of knee arthroscopy performed for residents aged 50 or over in SWSLHD divided by the number of SWSLHD residents aged 50 or over). Results for NSW and districts include patients aged 50 years and over, taking into account patient flows between districts (inflow: patients lived in other LHDs received surgery in this LHD’s public hospitals; outflow: patients lived in this LHD received surgery in other LHD’s public hospitals). The reduction in knee arthroscopy in both public and private hospitals between 2011 and 2015 for each district was assessed in order to capture any reduction since the first implementation in 2011 (Additional file 1: Table S3 includes an update of the complete data in 2015 and the reduction up to June 30, 2017).

Reductions in knee arthroscopy at four SWSLHD ‘intervention’ hospitals were further examined by a controlled before and after study design using a GEE Poisson model. Due to a possible lag in uptake of the recommendation and the fact that waiting lists were not altered, we chose 1st January of the year following the intervention to be the cut-off between pre- and post-implementation. Therefore, the post intervention period for three hospitals started on January 1, 2013 and the last one started at January 1, 2014. Earlier time points (January 1, 2012 and July 1, 2012) were used in sensitivity analyses. Trends in the number of arthroscopies before and after the policy change were compared with other matched control hospitals (one intervention hospital matched with four controls). All hospitals included in this part of the analysis are acute public hospitals. Control and intervention hospitals were also matched for the total number of knee arthroscopies between January 1, 2008 and December 31, 2012 (the fourth hospital was matched based on January 1, 2009 and December 31, 2013). All arthroscopies performed by September 30, 2016 were included in the follow-up analyses. The GEE Poisson model was used to examine arthroscopy volumes after the policy change, taking into account different follow-up times at each intervention hospital. Analyses are based on five years of data preceding the policy change and 45 months of follow-up data for three hospitals and 33 months for the remaining hospital. Due to a small number of intervention hospitals (*n* = 4), model-based standard errors were used to assess the significance of the findings. In the GEE analysis, the regression coefficient (β) represents the log-scale of the outcome variable so that the reduction rate at intervened hospitals is given by 1-e^β^. A negative value for β indicates that the rates for knee arthroscopy decreased following intervention, and vice versa for a positive value for β. This is also known as difference in difference regression which is often used to estimate the effect of a specific intervention or treatment by comparing the changes in outcomes over time between the intervention group and the control group. The analyses were performed using SAS software, version 9.4 (Cary, NC) [25].

## Results

Between 2004 and 2016, there were more than 10,000 knee arthroscopies performed on people aged 50 years or over in NSW each year of the study period (Data is provided in Additional file 1: Table S2).

**Fig. 1:**
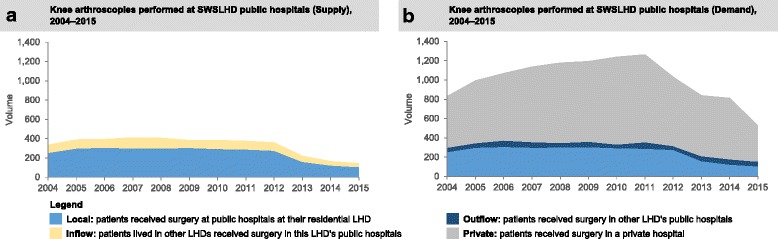
Knee arthroscopy (**a**) supply and (**b**) demand, for age 50+ years in SWSLHD between 2004 and 2015

**Fig. 2:**
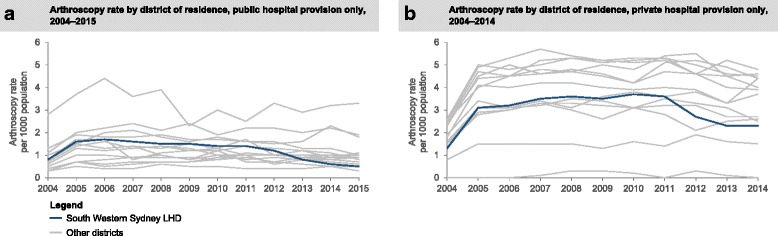
Knee arthroscopy demand rates for age 50+ by districts, (**a**) public and (**b**) private, between 2004 and 2015

Examining the knee arthroscopy volumes in the intervention district, by the amount of arthroscopies provided by public hospitals within the district (Supply) and the total arthroscopies performed on the district residents, regardless of where they received surgery (Demand), there was a clear drop after 2011 (Fig. 1a and b). The reduction in arthroscopies performed on SWSLHD residents was not only seen in public hospitals but also the surgeries performed in the private sector, including residents treated in private hospitals at other LHDs (Fig. 1b). A 40% or greater decrease in rates of knee arthroscopy in public sector was observed in five of 15 districts over the study period (Fig. 2a). In the period 2011–15, the largest decrease (from 1.4 to 0.5 per 1000 population) was found in the intervention district (SWSLHD). A similar drop in private sector was observed in SWSLHD between 2011 and 2014, from 3.6 to 2.3 per 1000 population, which was also the largest reduction among all districts in NSW (Fig. 2b). In view of the overall decrease noted, we calculated the overall (state-wide) decrease in the rate of knee arthroscopy in people aged 50 years or older using the most recent data available (Additional file 1: Table S3). This showed a decrease from a peak of 5.4 per 1000 people in 2011 to 2.8 per 1000 people in the 12 months up to June 30, 2017, a decline of 48% over 4.5 years.

Table 1 presents the reduction from 2011 to 2015 for NSW overall and by district. Between 2011 and 2015, the number of knee arthroscopies in NSW reduced by 39% (95% CI: 38–40%). Considering arthroscopies in both public and private hospitals by district, a clear reduction in arthroscopies (range from 7% to 58%) was shown for all districts except for Far West (number of arthroscopy in Far West LHD increased from 31 to 41 over the same period). The reduction in SWSLHD was the highest among all districts (58%, 95% CI: 55–62%) (Table 1).

**Table 1:** Reductions in numbers of knee arthroscopies for age 50+ between 2011 and 2015 by district, public and private hospitals, NSW

NSW district	Arthroscopy surgeries, 12 months to December 31, 2011	Arthroscopy surgeries, 12 months to December 31, 2015^b^	Reduction	Reduction percentage (%)	95% CI	
Central Coast	792	430	362	46%	42%	49%
Far West	31	41	−10	−32%	−14%	−50%
Hunter New England	2021	1554	467	23%	21%	25%
Illawarra Shoalhaven	497	370	127	26%	22%	29%
Mid North Coast	735	516	219	30%	26%	33%
Murrumbidgee	438	383	55	13%	9%	16%
Nepean Blue Mountains	601	354	247	41%	37%	45%
Northern NSW	476	409	67	14%	11%	17%
Northern Sydney	2012	905	1107	55%	53%	57%
South Eastern Sydney	2133	1187	946	44%	42%	46%
South Western Sydney	899	374	525	58%	55%	62%
Southern NSW	133	124	9	7%	2%	11%
Sydney	659	325	334	51%	47%	55%
Western NSW	390	254	136	35%	30%	40%
Western Sydney	841	541	300	36%	32%	39%
NSW overall	12,703	7786	4917	39%	38%	40%

^a^Figures included knee arthroscopies in both public and private hospitals; St Vincent district data is not presented due to confidentiality issue

^b^Due to incomplete private hospital data after July 1, 2015 at the time of this analysis, the number of knee arthroscopy in private hospitals during 2015 is underestimated

Table 2 summarised the number of knee arthroscopy before and after the policy implementation at both intervention and control hospitals. For example, in the first intervention hospital, there were 102 and 53 knee arthroscopies performed before and after the intervention, respectively. The corresponding figures in control hospitals were, in average, 104 and 87. It was clear that the number of knee arthroscopy reduced after the intervention at both intervention and control hospitals, despite the reduction scale at intervention hospitals seemed to be greater than that at control hospitals. Tables 3 present the estimation of reductions in knee arthroscopies in four hospitals where the policy was implemented. The number of knee arthroscopy at four intervention hospitals reduced by 56% (95% CI: 11%–79%, *p* = 0.02). Sensitivity analyses adjusting cut off dates resulted in similar findings.

**Table 2:** The number of knee arthroscopy pre- and post- intervention at intervention and control hospitals (public only)

Policy		Intervention 1	Control 1	Intervention 2	Control 2	Intervention 3	Control 3	Intervention 4	Control 4	All interventions (*n* = 4)	All control (*n* = 16)
Before^a^	Sum	102	415	595	2080	410	1539	142	612	1249	4646
	Average	102	104	595	520	410	385	142	153	312	290
After	Sum	53	349	138	1181	133	1040	40	371	364	2941
	Average	53	87	138	295	133	260	40	93	91	184
	Follow up Period (months)	45	45	45	45	45	45	33	33	39	39

^a^Both Intervention and control hospitals have 5 year pre intervention data; the number of knee arthroscopy are rounded

**Table 3:** Model estimations of reduction in knee arthroscopy in intervention hospitals (vs. control hospitals^a^, public only)

	Point estimates	95% CI		*p*-value	Incidence rate ratio
Intervention	0.08	−0.23	0.38	0.630	
Time	−0.13	−0.40	0.13	0.323	
Intervention ^a^ Time	−0.82	−1.52	−0.12	0.022	0.44 (0.21–0.89)

^a^Control hospitals were matched by volume of knee arthroscopy, and one intervention matches with four controls

## Discussion

Our results provide empirical evidence of practice change in response to an evidence-based policy in SWSLHD. The rate of knee arthroscopy in patients aged 50 years or over in SWSLHD decreased by almost 60% between 2011 and 2015 following the policy change. Despite an overall reduction over time seen at state level, the reduction was significantly larger in the intervention LHD. The results of difference in difference regression also found intervention hospitals had statistically significant more reductions than control hospitals. It is worthwhile to mention the fact that the intervention in the intervention hospital was not a complex intervention requiring heavy investments. It was a simple clinical governance and education process that most probably generated a reconsideration of the indication for arthroscopies and referral to surgeons. This study provide empirical evidence to show a very simple process worked, and therefore, this could be scaled and potentially tried for other surgical procedures that may not be warranted in all patients.

Although a reduction in rates of knee arthroscopy has been warranted, there has previously been little evidence of a significant fall in rates [26]. In the United States, both increase and decrease in the knee arthroscopies were found [6, 27] and the trends are not consistent with a recent study showing the annual rate of knee arthroscopy dropped between 1998 and 2002 but increased between 2006 and 2010 [28]. Studies from UK and Canada showed a reduction from 1993 to 2004 [29]. Furthermore, Mattila et al. [30] reported recent reductions in knee arthroscopy rates in Sweden and Finland, which could be attributed to well-established scientific evidence against knee arthroscopy for osteoarthritis. A recent study found there was a reducing trend in meniscus surgeries in the Netherlands following a nationwide guideline and the incidence seemed to increase in the last year of the study period [31].

Due to evidence showing that there was great variation between hospitals in patients with knee osteoarthritis receiving knee arthroscopy in Australia [32], and evidence against the effectiveness of knee arthroscopy, some clinicians at SWSLHD initiated a policy in 2011 to restrict knee arthroscopy for patients 50 years and over, regardless of the presence of knee osteoarthritis. Further steps have been taken in this district to reduce the rates of inappropriate knee arthroscopy by educating primary care providers, in order to reduce referrals and unnecessary investigations (MRI scans) for people with knee pain. Our initial findings demonstrated that clinician led policy change, based on high quality evidence is associated with a reduction in inappropriate surgery.

The policy under study was adopted by four public hospitals in SWSLHD in 2011–2013; however a drop in volumes was also observed over the study period in the other hospitals in the district as well as in other districts and in NSW overall. The fall in knee arthroscopy rates after 2011 observed in various districts suggests some practice change in response to the evidence. However, it was not possible to determine the extent to which the reduction observed in the intervention district was a result of the policy change. The interpretation of rate reduction observed should be cautious as rates were estimated based on “demand” rather than “supply” (no fixed denominator). The reduction in rate based on “demand” reflects the change in need for knee arthroscopy for residents in this particular LHD. This is because “demand” includes “outflow” as it measures surgery on patients living within an LHD, regardless of where they have surgery. Supply data reflects the number of surgeries performed in this LHD, regardless of where the patient lives. By using demand (and including outflow), we have addressed the criticism that the reduction in rates of surgery was due to patients seeking treatment outside the LHD.

A controlled before and after study design was used to assess the cause-effect of the policy implementation at the four intervention hospitals (within the intervention district). Four control hospitals from other LHDs were matched with each intervention hospitals in terms of hospital type (public), acute hospital and total volume of knee arthroscopies performed in the 5 years before the policy implementation.

The results showed that compared with hospitals without the policy, intervention hospitals reduced the number of knee arthroscopy by 56%. This reduction figure echoes the observation comparing the number of knee arthroscopy supplied by LHDs, which found that district of the intervention had the greatest reduction (58%) among all districts. Despite a small sample size, the reductions found comparing with control hospitals were statistically significant, suggesting the success of the policy. The reduction translates to 26 less cases performed per year in each hospital between 2012 and 2015 (95% CI: 22–30). Over the study period, we did not observe significant change in knee replacement rate for SWSLHD residents in the same age group (data not shown). The longer trend of knee replacement may need to be re-examined to ensure the reduction in knee arthroscopies does not lead to an increase in knee replacement surgery. This study is strengthened by using administrative datasets covering the whole state, which allowed comprehensive coverage and allowed measurement of district inflow and outflow and diversion to private hospitals. It also allowed measurement of the lead-in period prior to the policy change. The quasi-experimental design with control group enabled to examine the effect of the policy despite the overall reduction in arthroscopies in NSW.

Evidence on behavioural interventions that reduce inappropriate practice can be found [33–35] but not specifically for knee arthroscopy. From the review of interventions that reduce inappropriate transfusion, the results from the pre- and post-intervention, including education, retrospective audit, prospective audit along with a new request form, patient-specific decision support or new transfusion algorithm, have shown some reductions in inappropriate transfusion [34]. Different definitions of inappropriateness may make comparison difficult, but Wilson et al. concluded that there is evidence to support the possibility of behavioural interventions to change inappropriate practice among health care providers [34].

It is not possible to determine the relative contribution of the administrative intervention (department head approval) and the letter sent to general practitioners in the district.

A limitation of the study is that it did not measure ‘leakage’ (or patient flows) across state borders. This mainly affects the few districts that are adjacent to other states. Due to incomplete private hospital data in 2015, the overall reduction might be overestimated, but this should not influence the comparison between LHDs. Additional file 1: Table S3, with the complete data in 2015 and the overall reduction in arthroscopies between the 12 months to December 31, 2011 and the 12 months to June 30, 2017, consolidates our findings. While a reduction in private sector arthroscopies was observed in SWSLHD between 2012 and 2014, we could not properly assess the significance of reductions in the private sector as we have limited information to differentiate which private hospitals were influenced by the policy. Similarly, some clinicians at ‘intervention’ hospitals may work at private hospitals at other LHDs. Due to the control groups not being selected randomly, selection bias may also exist [36]. We have not adjusted for the number of orthopaedic surgeons in each department or the surgical volume of the department but these variables may not be associated with the timing or extent of practice change. In addition, the sample size maybe too small to limit the ability to generalise the findings to all hospitals in NSW or other countries. Nevertheless, the results from comparison between districts and to external controls showing similar reductions support the effect of the policy on reducing the knee arthroscopies despite the overall reduction in NSW.

## Conclusion

This is the first study in Australia to report a decrease in knee arthroscopy as a result of clinician-led policy change, which involved a simple clinical governance process and education letter. This study shows a significant reduction in the rate of knee arthroscopic surgery in patients aged 50 years and over after local implementation of the policy, despite an overall reduction in NSW since 2011. While evidence in this study generally supports the effect of the policy in reducing knee arthroscopies in the four hospitals and potentially in the whole district, a future assessment with more participating hospitals with sufficient follow-up period of data is recommended to verify the causal effect of the policy.

## Additional file

Additional file 1: **Table S1.** Knee arthroscopy procedures with numbers from 2004 to 2016, inclusive for age 50+. **Table S2.** Number of knee arthroscopies and total knee replacement (age 50 or over) in NSW between 2004 and 2016. **Table S3.** Reductions in numbers of knee arthroscopies for age 50+ between 2011 and June 30, 2017 by district, public and private hospitals, NSW. (DOCX 18 kb)

## Abbreviations

GEE: Generalised estimating equations
LHD: Local Health District
NSW: New South Wales
SWSLHD: South Western Sydney Local Health District
