# Supplementary material for: A controlled before-after study to evaluate the effect of a clinician led policy to reduce knee arthroscopy in NSW
Source: BMC Musculoskelet Disord. 2018 May 16;19:148. doi: 10.1186/s12891-018-2043-5 (PMC5956807; doi:10.1186/s12891-018-2043-5)
Supplement: Supplementary file 1 — Table S1. Knee arthroscopy procedures with numbers from 2004 to 2016, inclusive for age 50+. Table S2. Number of knee arthroscopies and total knee replacement (age 50 or over) in NSW between 2004 and 2016. Table S3. Reductions in numbers of knee arthroscopies for age 50+ between 2011 and June 30, 2017 by district, public and private hospitals, NSW. (DOCX 18 kb) [file 12891_2018_2043_MOESM1_ESM.docx]

Table S1 Knee arthroscopy procedures with numbers from 2004 to 2016*, inclusive for age 50+

| ACHI Procedure codes | Procedure description | Public | Private | Total |
| --- | --- | --- | --- | --- |
| 49557-00 | Arthroscopy of knee | 156 | 105 | 261 |
| 49557-02 | Arthroscopic excision of meniscal margin or plica of knee | 4857 | 4898 | 9755 |
| 49558-00 | Arthroscopic debridement of knee | 1866 | 303 | 2169 |
| 49558-01 | Arthroscopic chondroplasty of knee | 3987 | 4488 | 8475 |
| 49560-03 | Arthroscopic meniscectomy of knee | 4295 | 1980 | 6275 |
| 49561-01 | Arthroscopic meniscectomy of knee with debridement, osteoplasty or chondroplasty | 20403 | 7384 | 27787 |
| 49562-01 | Arthroscopic meniscectomy of knee with chondroplasty and multiple drilling or implant | 66667 | 12590 | 79257 |
| 49503-00 | Meniscectomy of knee | 2296 | 72 | 2368 |
| Total |  | 104527 | 31820 | 136347 |

*Due to incomplete private hospital data after July 1, 2015 at the time of this analysis, the number of knee arthroscopy in 2015 and 2016 are underestimated; 2016 data only include public hospitals records up to September 30, 2016

Table S2 Number of knee arthroscopies and total knee replacement (age 50 or over) in NSW between 2004 and 2016

|  | 2004 | 2005 | 2006 | 2007 | 2008 | 2009 | 2010 | 2011 | 2012 | 2013 | 2014 | 2015^*^ | 2016^*^ |
| --- | --- | --- | --- | --- | --- | --- | --- | --- | --- | --- | --- | --- | --- |
| Surgery for under 50 | 9488 | 9532 | 9572 | 9276 | 9299 | 9082 | 9169 | 9487 | 8677 | 8438 | 8766 | 5759 | 1606 |
|  |  |  |  |  |  |  |  |  |  |  |  |  |  |
| Estimated population 50 or over | 2009016 | 2046955 | 2083731 | 2137050 | 2191233 | 2245565 | 2305219 | 2366855 | 2422210 | 2479458 | 2534431 | 2583934 | 2647470 |
| Surgery for 50 or over | 10391 | 10475 | 10880 | 11648 | 11865 | 11762 | 12137 | 12705 | 12119 | 11391 | 11387 | 7787 | 1414 |
| Private hospitals | 7853 | 7938 | 8283 | 9033 | 9161 | 9204 | 9525 | 9953 | 9574 | 8987 | 8984 | 5619 | 32 |
| Public hospitals | 2538 | 2537 | 2597 | 2615 | 2704 | 2558 | 2612 | 2752 | 2545 | 2404 | 2403 | 2168 | 1382 |
| Local^1^ | 2138 | 2129 | 2166 | 2148 | 2201 | 2129 | 2178 | 2223 | 2172 | 1980 | 2030 | 1821 | 1175 |
| Outflow^2^ | 397 | 398 | 421 | 456 | 493 | 423 | 428 | 527 | 370 | 420 | 371 | 346 | 205 |
| Total knee replacement for 50 or over | 7743 | 8918 | 9475 | 9585 | 10338 | 10879 | 12167 | 13062 | 13909 | 14357 | 15021 | 12570 | 4312 |

*Due to incomplete private hospital data after July 1, 2015 at the time of this analysis, the number of knee arthroscopy in 2015 and 2016 are underestimated; 2016 data only include public hospitals records up to September 30, 2016

1: Local: patients received surgery at public hospitals at their residential LHD

2: Outflow: patients received surgery in other LHD’s public hospitals

Table S3 Reductions in numbers of knee arthroscopies for age 50+ between 2011 and 30^th^ June 2017 by district, public and private hospitals, NSW

| NSW district | Arthroscopy surgeries, 12 months to December 31, 2011 | Arthroscopy surgeries, 12 months to December 31, 2015 | Arthroscopy surgeries, 12 months to June 30, 2017 | Reduction between December 31, 2011 and June 30, 2017 | Reduction percentage (%) | 95%CI | |
| --- | --- | --- | --- | --- | --- | --- | --- |
| Central Coast | 792 | 511 | 424 | 368 | 47% | 43% | 50% |
| Far West | 31 | 41 | 34 | -3 | -10% | -22% | 2% |
| Hunter New England | 2021 | 1665 | 1176 | 845 | 42% | 40% | 44% |
| Illawarra Shoalhaven | 497 | 474 | 347 | 150 | 30% | 26% | 34% |
| Mid North Coast | 735 | 516 | 458 | 277 | 38% | 34% | 41% |
| Murrumbidgee | 438 | 513 | 394 | 44 | 10% | 7% | 13% |
| Nepean Blue Mountains | 601 | 564 | 433 | 168 | 28% | 24% | 32% |
| Northern NSW | 476 | 419 | 362 | 114 | 24% | 20% | 28% |
| Northern Sydney | 2012 | 1495 | 1281 | 731 | 36% | 34% | 38% |
| South Eastern Sydney | 2133 | 1345 | 1034 | 1099 | 52% | 49% | 54% |
| South Western Sydney | 899 | 507 | 401 | 498 | 55% | 52% | 59% |
| Southern NSW | 133 | 124 | 120 | 13 | 10% | 4% | 15% |
| Sydney | 659 | 415 | 300 | 359 | 54% | 51% | 58% |
| Western NSW | 390 | 264 | 268 | 122 | 31% | 27% | 36% |
| Western Sydney | 841 | 700 | 566 | 275 | 33% | 29% | 36% |
| **NSW overall** | **12703** | **9572** | **7620** | **5083** | **40%** | **39%** | **41%** |
| **NSW overall rate (per 1,000 of over 50 population)** | **5.4** | **3.7** | **2.8** | **2.6** | **48%** | - | |

*figures included knee arthroscopies in both public and private hospitals; St Vincent district data is not presented due to confidentiality issue.
